# Comment on Balwierz et al. Potential Carcinogens in Makeup Cosmetics. *Int. J. Environ. Res. Public Health* 2023, *20*, 4780

**DOI:** 10.3390/ijerph20196901

**Published:** 2023-10-09

**Authors:** Ishrat Chaudhuri, Yufanyi Ngiewih, Len Levy, Robert J. McCunney

**Affiliations:** 1 Cabot Corporation, Billerica, MA 01821, USA; 2 Orion Engineered Carbons GmbH, 65760 Eschborn, Germany; yufanyi.ngiewih@orioncarbons.com; 3 School of Water, Energy and Environment, Cranfield University, Cranfield MK43 0AL, UK; len.levy@cranfield.ac.uk; 4 Pulmonary Division, Brigham and Women’s Hospital, Harvard Medical School, Boston, MA 02215, USA; bobmccunney@gmail.com

We read with interest the article by Balwierz et al. [1] in which the authors discuss potential carcinogens that may be present in cosmetics. Various inaccuracies and misrepresentations of the science related to carbon black, however, are present in the article, which we would like to note.

The authors incorrectly define carbon black in stating that “*Carbon black (charcoal) is a black-colored substance produced from wood through a dry distillation process*”. This statement is incorrect. To the contrary, carbon black (CAS number 1333-86-4) is not charcoal, nor is it produced from wood; instead, it consists of nearly pure elemental carbon (upwards of 98%) and is manufactured via either the partial combustion or thermal decomposition of gaseous or liquid hydrocarbons under controlled conditions. This process is optimized to yield a variety of grades having specified ranges of properties (e.g., specific surface area, particle size and structure, conductivity, and color) [2,3].

Furthermore, the authors interchangeably and erroneously use black carbon or carbon as synonyms for carbon black. Unfortunately, this mischaracterization of carbon black is not uncommon in the scientific literature [4,5]. Other forms of carbon are often mistakenly referred to as carbon black; these include black carbon (or soot) and activated carbon (or charcoal). The former is an undesired carbonaceous byproduct of the incomplete combustion of fossil fuels and biomass, and the latter is manufactured from the thermal decomposition of organic material with a high carbon content (such as wood, peat, or coconut shells). This confusion in the literature has prompted other authors to clarify the situation and describe the differences between other forms of carbon and carbon black [4,5,6].

The authors rely on the International Agency for Research on Cancer (IARC) classification for carbon black for their designation of carbon black as a carcinogen. (We also note that in the abstract, the authors have confused the IRAC (Insecticide Resistance Action Committee) with the IARC (International Agency for Research on Cancer). It is particularly important that information given in abstracts be accurate as this information is often what is accessible to readers.) Various caveats associated with carbon black’s classification by the IARC as a Group 2B carcinogen (*possibly carcinogenic to humans*) should be noted. (The authors incorrectly state that the IARC classified carbon black as a “probable” carcinogen, a term that applies to IARC Group 2A carcinogens.) The 2B classification for carbon black is based on the IARC criteria of “sufficient evidence in experimental animals,” but “inadequate evidence in humans” [7]. It is important to note that lung tumors following carbon black inhalation have only been observed in rats and not in other rodent species such as mice and hamsters. Similar findings have not been noted in humans. Scientific evidence indicates that the laboratory rat is a uniquely sensitive species in its pulmonary responses to persistent high doses of inhaled low-solubility particles causing “lung overload” [8]. Recently, an international scientific panel of experts in the fields of chemicals regulation, toxicology, epidemiology, and particle science concluded that rat lung cancer occurring only under conditions of lung particle overload, in the absence of corroborating data from other species, should not be interpreted as a cancer hazard for humans [9]. Carcinogenicity has only been observed in rats following inhalation at lung overload doses and not through the oral or dermal routes. Epidemiological studies on carbon-black-manufacturing workers do not show an association between carbon black inhalation exposure and elevated lung cancer rates [10]. The IARC concluded that “*In several experiments of dermal application in mice that used various carbon blacks, no carcinogenic effect on the skin was observed*” [7].

The authors misinterpret some of the epidemiological evidence regarding carbon black in the following statement: “Regular inhalation of carbon black may contribute to reduced lung functioning in humans, as confirmed by epidemiological studies by Puntoni (reference number cited in Balwierz et al.: [112]) and Wellmann (reference number cited in Balwierz et al.: [78]).” Neither of these references, however, note reduced lung function in humans. Puntoni et al.’s work is a case–control study on bladder cancer, and Wellman et al.’s work is a cohort mortality study; neither of these publications addressed lung function in humans. 

A further misinterpretation of the epidemiological literature follows in this statement: “Epidemiological studies involving workers having occupational contact with carbon black by inhalation and dermal routes had an increased incidence of excessive dermal keratosis, as well as leukoplakia. These lesions represent known conditions that can predispose to the formation of cancerous foci, so-called precancerous conditions (reference numbers cited in Balwierz et al.: [79,80,113]).” The authors, however, have misinterpreted the studies they cited in the previous comment, as none of them describe keratosis and/or leukoplakia. In contrast, Boland et al. [79] addressed differences in the mechanisms of toxicity of carbon black and titanium dioxide; Valberg et al. [80] analyzed the discordance between rat studies and human epidemiology studies; and Yong et al. [113] provided a meta-analysis of carbon black worker mortality studies.

Balwierz et al. state that “According to the available literature, when used long term, the carbon black in makeup cosmetics applied around the eyes (mascara and pencils) causes reddening of the eyelid conjunctiva”; however, a statement such as this warrants an appropriate reference. On the contrary, various regulatory and scientific bodies have reviewed scientific data on carbon black and have concluded that carbon black, with certain purity criteria, is safe for use in specific cosmetics. For example, the Scientific Committee on Consumer Safety (SCCS), which advises the European Commission on the health and safety risks of non-food consumer products in the European Union, has carried out a comprehensive risk assessment of carbon black. The SCCS adopted the opinion that the use of carbon black, with certain purity restrictions, in cosmetics is safe and poses no hazard risk to consumers in cosmetic products applied to healthy, intact skin. A separate opinion, developed for carbon black in its nano-structured form (with a primary particle size of 20 nm or larger), concluded that at concentrations of up to 10% *w*/*w* (weight in weight) as a colorant in cosmetic products, carbon black does not pose a risk of adverse effects in humans after application on healthy, intact skin [11]. Based on the SCCS’s opinions, the European Commission subsequently adopted a regulation (https://eur-lex.europa.eu/legal-content/EN/TXT/PDF/?uri=CELEX:32016R1120&from=EN, accessed on 10 April 2023), authorizing the use of carbon black as a colorant in cosmetics and amending Annex IV of the Cosmetic Regulation (Regulation (EC) No 1223/2009). In the US, the Food and Drug Administration (FDA) conducted an extensive safety evaluation on carbon black and determined that with certain purity restrictions (listed as D&C Black No. 2), it may be safely used for coloring the following cosmetics in amounts consistent with current good manufacturing practices: eyeliner, brush-on-brow, eye shadow, mascara, lipstick, blushers and rouge, makeup and foundation, and nail enamel (https://www.ecfr.gov/current/title-21/chapter-I/subchapter-A/part-74/subpart-C/section-74.2052, accessed on 10 April 2023). The purity criteria adopted for carbon black in the EU and US mainly regulate the impurity content of carbon black as it relates to polycyclic aromatic hydrocarbons (PAHs), heavy metals, and ash (the specific restrictions are listed in the references cited above).

Carbon black is listed by the IARC as a Group 2B carcinogen. However, it is important to note that this classification is based on studies on rats following inhalation at lung overload doses; there is no scientific evidence of carcinogenicity through the oral or dermal routes. Consequently, in the EU, the use of carbon black in sprayable cosmetic products that might lead to the exposure of consumers’ lungs via inhalation is disallowed. The evidence on carbon black indicates that when used in ways that are sanctioned by regulatory bodies or carbon black manufacturers, i.e., in cosmetics to be applied to healthy, intact skin, carbon black is safe. This position is consistent with opinions provided by the US FDA and the EU SCCS. 
